# A Preliminary Study to Evaluate the Immune Response to a Booster Dose of the Adult Tetanus-Diphtheria Vaccine (Td) Available in Iran

**DOI:** 10.5812/ijpr-146572

**Published:** 2024-09-11

**Authors:** Sepideh Noorian, Negar Mottaghi-Dastjerdi, Mohammad Soltany-Rezaee-Rad, Hamed Montazeri, Masoumeh Baghaei, Mohammad-Javad Niazi

**Affiliations:** 1 Department of Pharmacognosy and Pharmaceutical Biotechnology, School of Pharmacy, Iran University of Medical Sciences, Tehran, Iran; 2 Behestan Innovation Factory, Tehran, Iran; 3 Department of Pharmaceutics, Faculty of Pharmacy, Tehran University of Medical Sciences, Tehran, Iran; 4 Department of Pharmaceutical Biotechnology, Faculty of Pharmacy, Kerman University of Medical Sciences, Kerman, Iran

**Keywords:** Tetanus, Tetanus Vaccine, Seroconversion, Booster Dose, Hyper-Immune, ELISA

## Abstract

**Background:**

Despite the availability of tetanus-diphtheria (TD) vaccines in Iran, the seroconversion rate of these products as a booster dose is unknown.

**Objectives:**

This study evaluates the seroconversion rate of the Iranian Td vaccine in adults who have not been vaccinated in the past decade.

**Methods:**

In this study, 20 adult volunteers aged 18 to 60 who had not received the Td vaccine in the past decade received a booster dose of the Iranian Td vaccine. Twenty-eight days after vaccination, the seroconversion rate was evaluated using the ELISA method. Vaccine adverse events were monitored at three time points after vaccination.

**Results:**

Seroconversion rates with the Iranian Td vaccine boosters were 75% and 90%, respectively, based on a 4-fold increase in anti-tetanus toxoid antibody titers and a 2- and 4-fold combination. Significant associations were found between the demographic data, specifically female gender and age 43 years and older, with seroconversion rates. Injection-site pain was the most common adverse reaction, with a frequency of 35%. No adverse events were reported between one week and one month after vaccination.

**Conclusions:**

Results showed that a booster dose of the Iranian Td vaccine produced a protective titer (> 0.1 IU/mL) and a long-term protective titer (> 1.0 IU/mL) in 100% of participants. The seroconversion rate of the Iranian Td vaccine was comparable to other common tetanus vaccines, including Tenivac^®^, Adacel^®^, Infanrix^®^, Tetavax^®^, and Vacteta^®^. The proportion of suitable candidates for plasma donation, based on minimum (2 IU/mL) and maximum (10 IU/mL) anti-tetanus toxoid antibody titers, was 100% and 45%, respectively.

## 1. Background

A potent neurotoxin produced by the spore-forming bacterium Clostridium tetani, which grows in an anaerobic environment, is the cause of tetanus, an acute, preventable disease that results in instability of the motor and autonomic nervous systems. C. tetani spores can infect wounds and abrasions and are widespread in soil and surrounding areas (1-3).

Despite a mortality rate of 50%, particularly in low- and middle-income countries, tetanus remains a global health problem. Tetanus vaccine development has progressed rapidly, leading to fewer outbreaks and a significant decline in the incidence of the disease in developed countries. Although tetanus is relatively uncommon, it is still a vaccine-preventable disease in most developed countries. In rare cases, a person can become immune to tetanus naturally or through the placenta. The only way to prevent tetanus is through vaccination with vaccines that contain tetanus toxoid (TT), which produces antibodies that neutralize the neurotoxin known as tetanospasmin (4-7).

Booster vaccination for adults and adolescents is essential for maintaining lifelong immunity against tetanus. Vaccine-preventable diseases such as tetanus can be controlled with timely booster doses (8, 9).

Tetanus toxoid is a monovalent vaccine or a component of combined diphtheria-pertussis-tetanus (DPT) or diphtheria-tetanus (DT) vaccines. In most countries, a monovalent vaccine or a combination vaccine with tetanus toxoid, such as Td and Tdp vaccines, is recommended as a booster dose every 10 years for the immunization of adults who completed tetanus vaccination in childhood, unless previously used for prophylaxis as part of wound treatment (10).

In Iran, vaccination against diphtheria, tetanus, and pertussis has been carried out since 1950 with a local vaccine produced by the Razi Institute. Previous studies have confirmed the effectiveness of the vaccine (11).

## 2. Objectives

The tetanus-diphtheria (Td) vaccine is available in the Iranian healthcare system, but the seroconversion rate of this product as a booster dose is unknown. This preliminary study aimed to evaluate the seroconversion rate of the Td vaccine available in Iran as a booster dose in healthy adult volunteers aged 18 to 60 years who have not received the Td vaccine in the past decade. The results of this preliminary study could be used in future research as a basis for pharmaceutical companies producing tetanus hyperimmune serum, determining if it is possible to use the serum of individuals who have received a booster dose of the Iranian Td vaccine (after 10 years) for this purpose. Side effects are also reported in this study; however, the small sample size prevents reliable determination of their prevalence.

## 3. Material and Methods

This study was designed as a before-and-after study and was conducted from March 2021 to September 2021. The study protocol was reviewed and approved by the Biologic Sampling Ethics Committee at Iran University of Medical Sciences (IUMS) (code of ethics: IR.IUMS.REC.1400.138 ) to ensure compliance with ethical standards. In addition to obtaining written consent forms, the study also adhered to ethical considerations by ensuring participant confidentiality through data anonymity. Participants were provided with clear information about the purpose, risks, and potential benefits of the study to make informed decisions. All participants in this preliminary study entered the study completely voluntarily and with full knowledge, and they could withdraw from the study at any time. To maintain ethical standards throughout the research process, continuous monitoring and periodic reviews were conducted.

Among the people who volunteered to participate in the study, those with Guillain-Barré syndrome, a history of allergy to vaccine components, or a history of any disease that might interfere with the study’s results were excluded. Additionally, individuals who participated in or planned to participate in a clinical trial with another investigational medicinal product within 30 days before the study vaccination or at any time during this study period, as well as within 4 weeks before or after another non-study-related clinical trial received or planned to receive a vaccine, were excluded. Pregnant women were also excluded from the study. The sample size was calculated using G-power software version 3.1 (Cohen's D = 0.8), and the sampling method was non-random. Finally, 20 healthy adults (18 - 60 years old) who had not received tetanus and diphtheria vaccines in the past 10 years and were willing to participate in this study were included.

After obtaining the written consent forms on day 1, history taking and physical examinations were conducted. Then, a 0.5 mL dose of the Td vaccine (Razi^®^) was administered intramuscularly into the deltoid region immediately after enrollment. The vaccine contains at least 5 Lf of Tetanus Toxoid and 2 Lf of Diphtheria Toxoid, with a maximum of 25.1 mg of aluminum phosphate based on aluminum ion as an adjuvant and 50 micrograms of thimerosal as a preservative.

To evaluate the seroconversion rate using commercial kits, tetanus titers were determined by enzyme-linked immunosorbent assay (ELISA). Participants were monitored for possible side effects for up to half an hour after booster vaccination at the vaccination site, one week later using a questionnaire, and one month later by a doctor.

According to previous studies, such as those on Tenivac^®^ and Adacel^®^, the criterion for a positive reaction to the vaccine was determined based on the pre-vaccination tetanus immunoglobulin level. For individuals whose pre-vaccination immunoglobulin level was 2.7 IU/mL or less, a four-fold increase in the antibody level was considered a positive reaction. For those whose pre-vaccination immunoglobulin level was more than 2.7 IU/mL, a doubling of the immunoglobulin level was counted as a positive reaction. Therefore, the difference between the two dependent average values accounts for a large number, based on the standardized effect size explanation. The effect size dz or the intensity of the effect can also be a large number.

To study the immune response to the booster dose of the tetanus Td vaccine available in Iran, serological examination was performed on the serum samples obtained from each subject before and 28 days after the booster vaccination. After collecting blood samples and storing them at room temperature for 30 minutes, the serum samples were separated by centrifugation at 4000 rpm for 10 minutes. Serum samples were stored at 2 – 8°C for 1 week and at -20°C until the end of the study.

The anti-tetanus immunoglobulin titer of the serum samples was measured with an ELISA test using the human Tetanus Toxoid IgG, Abnova^®^ kit (Taiwan Corporation), which has a measurement range of 0.01 to 5.0 IU/mL. Briefly, 100 µL of each diluted serum sample and standards were pipetted into the Tetanus antigen-coated wells. One well was left empty for a substrate blank. The plate was covered and incubated at room temperature for 60 minutes. After emptying the incubated solution, the plate was washed three times with 300 µL of diluted washing solution. Then, 100 µL of enzyme conjugate was pipetted into the wells. The covered plate was incubated at room temperature for 30 minutes. After discarding the incubated solution, the plate was washed three times with 300 µL of diluted washing buffer. Next, 100 µL of substrate solution, 3,3´,5,5´-tetramethylbenzidine (TMB), was pipetted into the plate, including the substrate blank. After covering the plate and incubating for 20 minutes at room temperature, 100 µL of TMB stop solution was pipetted into all wells, including the substrate blank, to stop the substrate reaction. After thoroughly mixing the plate, the optical densities (OD) were read at 450 nm. The raw data from the ELISA reader were analyzed using Gain-Data software (Arigo ELISA calculator). This software was used to draw the standard curve, and the regression model used was 4PL. The antibody concentrations in the samples were read from the standard curve determined from the standard samples.

According to WHO guidelines, antibody levels ≥ 0.1 IU/mL and > 1.0 IU/mL against tetanus were considered seroprotection (minimum protective titer) and long-term seroprotection, respectively. Additionally, multiple increases in antibody titers from pre-vaccination to post-vaccination were considered seroconversion rates for booster response (12).

Combined seroconversion in this study was defined as a 4-fold rise (SCR4) in antibody titer when the pre-vaccination tetanus antibody level was ≤ 1.0 IU/mL, and a 2-fold increase (SCR2) in antibody titer when pre-vaccination levels of tetanus antibodies were > 1.0 IU/mL, which does not need to reach a fourfold increase according to WHO requirements. The cut-off value of 1.0 IU/mL was chosen based not only on WHO requirements but also on previous studies of globally distributed vaccines, such as the Tdap vaccine (Infanrix^®^, GSK) (13).

Statistical analysis was performed using SPSS 26.0 software. Descriptive analyses were presented as percentages and means with standard deviation (SD) for quantitative variables and frequencies with percentages for qualitative variables.

Antibody levels were determined using the geometric mean concentration (GMC) of tetanus antibodies. Seroprotection and seroconversion rates were expressed as percentages and estimated with a 95% confidence interval (CI) in the study population. The booster response of antibodies was the proportion of participants who had a 4-fold (SCR4) or greater rise or a combination of SCR4 and SCR2 antibody rise post-vaccination, while the proportion of participants seroprotected (≥ 0.1 IU/mL) was considered the primary non-inferiority immunogenicity measure for tetanus antibodies. This study’s seroconversion rate was compared with those presented in similar studies involving Tenivac^®^, Adacel^®^, Tetavax^®^, Vacteta^®^, and Tdap (Infanrix^®^, GSK) vaccines (13-16). The non-inferiority criterion for seroconversion, based on previous studies and WHO guidelines, was considered to be less than a 10% difference between the upper limit of the 95% confidence interval of seroconversions (17).

To produce hyperimmune serum, different studies have reported varying antibody concentration thresholds, including concentrations of 2, 3, 4, 5, 8, and 10 IU/mL (18-22).

Mean changes in antibody concentrations before and after booster vaccination were analyzed using the paired *t*-test. One-way ANOVA tests were used for group comparisons when appropriate. Correlation analysis was performed using multivariate regression analysis to explore factors affecting antibody levels and to investigate variables predicting seroconversion rates. The level of statistical significance was set at P < 0.05.

Vaccine adverse events were monitored 1 hour, 1 week, and 1 month after vaccination.

**Figure 1.**
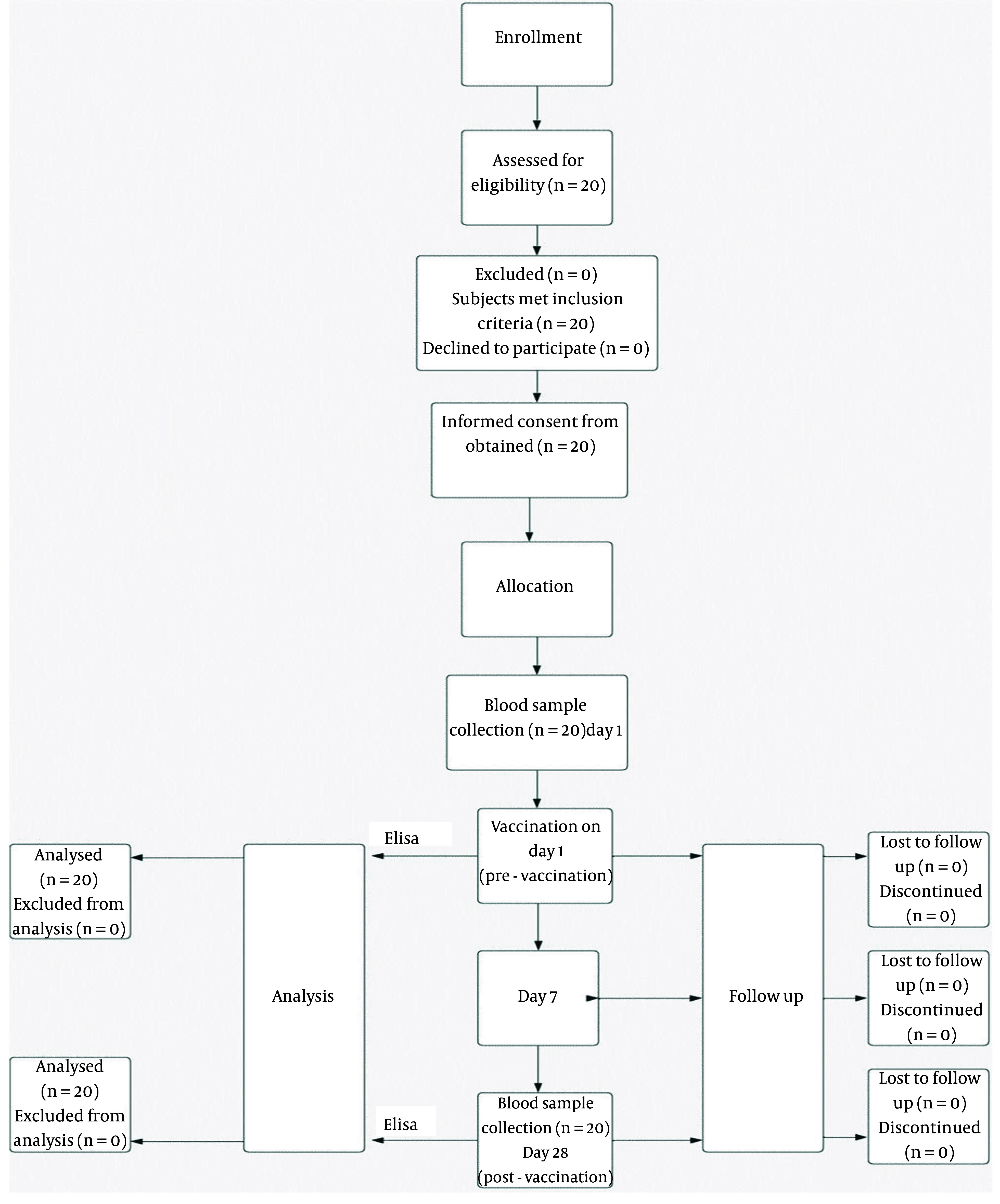
Summary algorithm of study methodology

A summary of the study methodology is shown in the algorithm in Figure 1. 

## 4. Results

A total of 20 enrolled subjects completed the study. The demographic characteristics of the subjects are described in Table 1. In this study, the period between the previous and current tetanus vaccinations was considered the pre-immunization period.

**Table 1.** Demographic Characteristics of the Study Population Expressed with Proportions or Means Including the 95% Confidence Interval ^a^

Characteristics	No.	Values
**Gender**		
Male	10	50 (27.2 - 72.8)
Female	10	50 (27.2 - 72.8)
**Medical history**		
Yes	3	15 (3.21 - 37.89)
No	17	85 (62.11 - 96.79)
**Concomitant medication**		
Yes	5	25 (8.66 - 49.1)
No	15	75 (50.9 - 91.34)
**Related adverse events**		
Yes	7	35 (15.39 - 59.22)
No	13	65 (40.78 - 84.61)
**Age (y)**		
25 - 30	5	27.4 ± 1.34 (26.2 - 28.6)
31 - 36	6	33.5 ± 2.07 (31.8 - 35.2)
37 - 42	5	39.8 ± 2.59 (37.5 - 42.1)
≥ 43	4	46 ± 0.82 (45.2 - 46.8)
**Height (cm)**	20	172.1 ± 16.11 (165 - 179)
**Weight (kg)**	20	78.3 ± 8.37 (74.6 - 82)
**BMI (kg/m** ^**2**^ **)**		
18.5 - 24.99	8	22.39 ± 1.4 (21.4 - 23.4)
25 - 29.99	7	26.8 ± 1.5 (25.7 - 27.9)
≥ 30	5	31.82 ± 2.41 (29.7 - 33.9)
**Pre-vaccination period (y)**		
10 - 14	8	12.38 ± 1.85 (11.1 - 13.7)
15 - 29	10	20.3 ± 3.97 (17.8 - 22.8)
≥ 30	2	34.5 ± 0.71 (33.5 - 35.5)

^a^ Values are expressed as proportion percentage (95% CI) or mean ± SD (95% CI).

The subjects were divided into four, three, and three groups according to age, BMI, and period before vaccination, respectively. Although this study was conducted on healthy adults, volunteers with concomitant diseases, such as impaired thyroid function (10%), reproductive system defects (10%), a history of seasonal allergies (5%), a history of seizures (5%), and hair loss (5%), were included in the study. These diseases were not expected to affect the study results. Monitoring after vaccination showed no observed or reported effects of the vaccination on their conditions.

Vaccine-related adverse events were reported in seven subjects, including three men and four women, within 1 hour of vaccination (Table 1). Local adverse events occurred in seven (35%) subjects, while systemic adverse events occurred in two (10%) subjects. Pain at the injection site was the most common local adverse reaction. Moderate lethargy, mild hand heaviness, dizziness, and sleep disturbances were the most common systemic side effects. All adverse events resolved within seven days without specific medical intervention. No serious adverse events were reported. No adverse events were reported one week and one month after vaccination.

The mean serum concentration of anti-tetanus antibodies was 2.01 IU/mL on day 1 before booster vaccination and 13.59 IU/mL on day 28 after vaccination. The mean changes in antibody concentrations before and after vaccination were analyzed using the paired *t*-test. The results showed that the increase from 2.01 to 13.59 IU/mL was statistically significant (P < 0.05). This indicates that anti-tetanus antibodies increased significantly in subjects after the tetanus booster vaccination.

**Figure 2.**
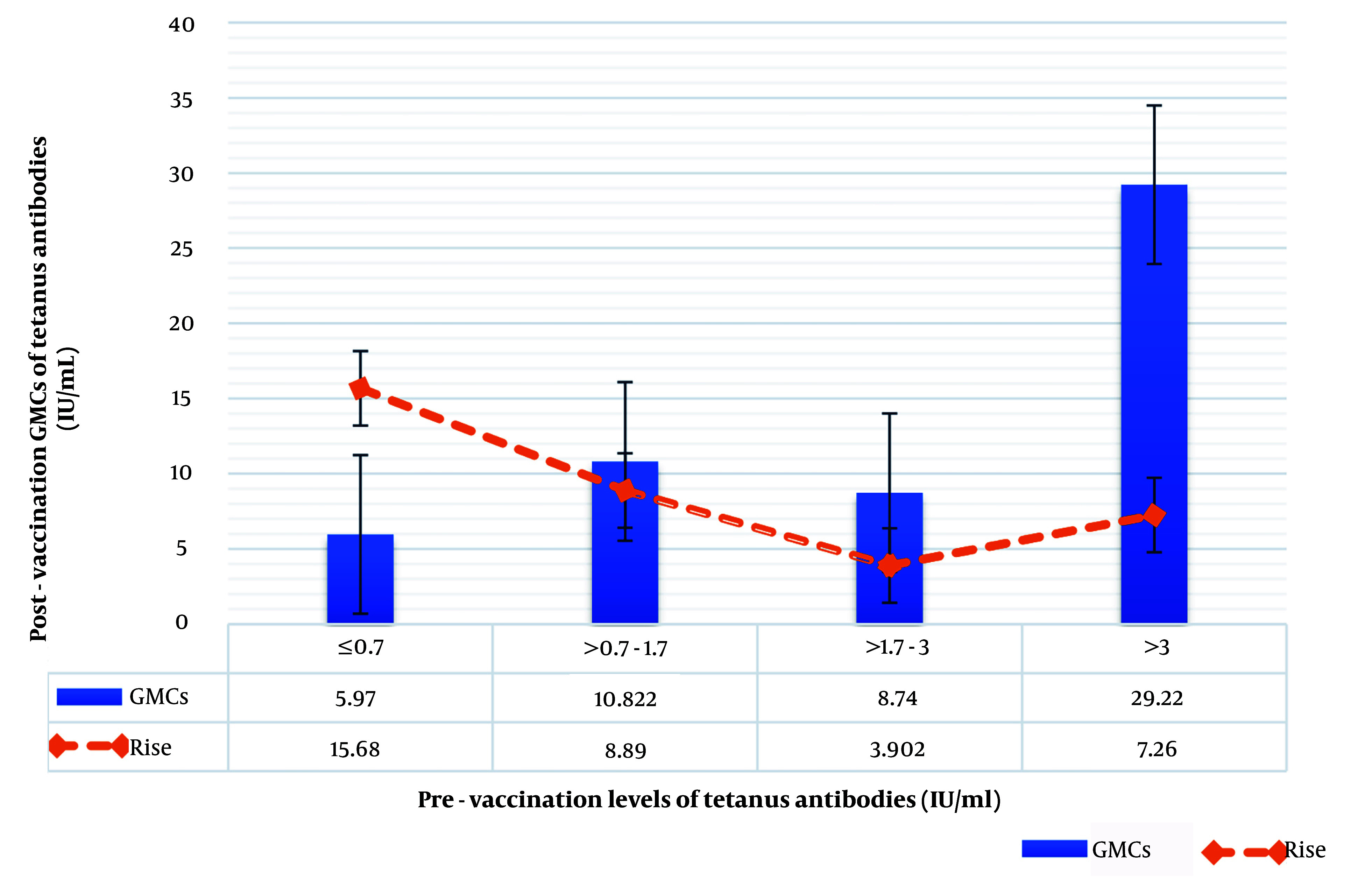
Geometric Mean Concentrations (GMCs) of tetanus antibodies and corresponding elevation in antibody levels after booster immunization, stratified by pre-vaccination tetanus antibody levels (IU/mL). The blue bars represent the GMCs of tetanus antibodies post-vaccination, while the orange dashed line indicates the relative increase in antibody levels. Error bars denote the standard error of the mean, illustrating the variability within each group. This figure highlights the dependency of antibody response on pre-vaccination antibody levels, showing both absolute antibody concentrations and the relative rise in antibody levels across varying baseline immunity categories.

The geometric mean increase in tetanus antibodies after booster vaccination (post-booster GMCs) was independent of adult sex, age, BMI, medical history, concomitant medications, associated adverse events, and pre-vaccination period. Furthermore, no statistically significant correlation was found between post-vaccination GMC and pre-vaccination GMC, expressed in an interquartile range: 0.7 IU/mL (25% quartile), 1.7 IU/mL (median), and 3 IU/mL (75% quartile), and the antibody increase after vaccination (Table 2 and Figure 2). 

**Table 2.** Post-booster Geometric Mean Concentrations (GMCs) of Tetanus Antibody and Pre to the Post-booster Level Ratio Including the 95% Confidence Interval

Variables	Post-Booster GMCs (95% CI) in IU/mL	P ^a^	Pre to the Post-Booster Level Ratio (95% CI)	P ^a^
**Gender**		0.551		0.243
Male	12.28 (6.27 - 18.3)		7.23 (3 - 11.5)	
Female	14.91 (7.02 - 22.8)		9.95 (4.7 - 15.2)	
**Medical history**		0.464		0.241
Yes	18.06 (3.06 - 33.1)		13.45 (1.85 - 25)	
No	12.80 (7.99 - 17.6)		7.73 (4.67 - 10.8)	
**Concomitant medication**		0.261		0.359
Yes	18.53 (6.23 - 30.8)		11.38 (3.91 - 18.9)	
No	11.95 (7.44 - 16.5)		7.66 (4.23 - 11.1)	
**Related adverse events**		0.513		0.928
Yes	11.30 (3.94 - 18.7)		8.81 (2.76 - 14.9)	
No	14.83 (8.81 - 20.9)		8.47 (4.67 - 12.3)	
**Age (y)**		0.257		0.897
25 - 30	10.81 (1.58 - 20)		8.414 (1.76 - 15.1)	
31 - 36	21.29 (11.3 - 31.3)		6.44 (4.01-8.87)	
37 - 42	9.99 (4.14 - 15.8)		9.62 (1.53 - 17.7)	
≥ 43	10.01 (4.62 - 15.4)		10.76 (2.51 - 19)	
**BMI (kg/m** ^**2**^ **)**		0.841		0.123
18.5 - 24.99	14.6 (7.14 - 22.1)		9.51 (4.47 - 14.6)	
25 - 29.99	14.3 (6.35 - 22.3)		4.47 (3.32 - 5.62)	
≥ 30	10.99 (1.71 - 20.3)		12.89 (4.61 - 21.2)	
**Pre-vaccination period (y)**		0.635		0.244
10 - 14	13.32 (5.11 - 21.6)		10.88 (4.72 - 17)	
15 - 29	15.22 (8.77 - 21.7)		5.61 (4.39 - 6.83)	
≥ 30	6.53 (4.47 - 8.59)		5.93 (2.42 - 9.44)	
**Pre-vaccination GMC (median) (IU/mL)**		0.055		0.270
≤ 1.7	8.88 (3.74 - 14)		11.60 (5.87 - 17.3)	
> 1.7	18.3 (11.5 - 25.1)		5.58 (3.94 - 7.22)	

^a^ Statistical significance calculated with the one-way ANOVA test.

The tetanus anti-toxoid levels (pre- and post-vaccination) and seroconversion or booster response rates (SCR4 and a combination of SCR4 and SCR2) after one dose of the Td vaccine available in Iran are presented in Table 3. 

**Table 3.** Pre-vaccination and Post-vaccination Antibody Responses and Booster Response Rates to Tetanus Toxoid Following Td Vaccine Available in Iran ^a^

Timing	Percent of Participants with Specified Level of Tetanus Anti-toxoid and Booster Response			
	≥ 0.1 IU/mL	≥ 1.0 IU/mL	Booster Response ^b^	Booster Response (SCR4 & SCR2) ^c^
**Pre-vaccination**	100 (83.16 - 100)	70 (45.72 - 88.11)		
**Post-vaccination**	100 (83.16 - 100)	100 (83.16 - 100)	75 (50.90 - 91.34)	90 (68.3 - 98.76)

^a^ Values are expressed as proportion percentage (95% CI).

^b^ Booster response: 4-fold increase in antibody levels.

^c^ Booster response: A combination of 4-fold and 2-fold rise in antibody levels; if pre-vaccination level ≤ 1.0 IU/mL, 4-fold increase (SCR4). If the pre-vaccination level > 1.0 IU/mL, a 2-fold increase (SCR2).

Before vaccination, all twenty subjects had an anti-tetanus antibody concentration ≥ 0.1 IU/mL, while only 70% (95% CI = 50 - 90) of them (eight men and six women) had an anti-tetanus antibody concentration ≥ 1.0 IU/mL. After receiving a tetanus booster dose, 100% of subjects achieved anti-tetanus antibody concentrations of ≥ 1.0 IU/mL.

SCR4, defined as at least a fourfold increase in antibody levels following booster administration, reached 75% (95% CI 50.9 – 91.34) in the entire study population (Table 3). A combination of SCR4 and SCR2, defined as at least a 4-fold increase in antibody levels after booster vaccination if the pre-vaccination value was ≤ 1.0 IU/mL and a 2-fold increase in antibody levels after booster vaccination if the pre-vaccination value was > 1.0 IU/mL, reached 90% (95% CI 68.3 - 98.76) in the study population (Table 3). 

A comparison of the seroconversion rates of the Iranian tetanus vaccine with those of Tenivac^®^, Adacel^®^, Tetavax^®^, Vacteta^®^, and Tdap (Infanrix^®^, GSK) vaccines is shown in Table 4. 

**Table 4.** Comparison of Seroconversion of Iranian Tetanus Vaccine with Tenivac^®,^ Adacel^®,^ Tetavax^®^ or Vacteta^®,^ and Tdap (Infanrix^®^, Gsk) (13-16) ^a^

Vaccines	Seroconversion, at Least a 4-fold Rise in Antibodies (SCR4)	Seroconversion (A Combination of SCR4 and SCR2)
**Iranian Tetanus vaccine**	75 (50.90 - 91.34)	90 (68.3 - 98.76)
**Tenivac** ^**®**^^**b**^	84 (78.7 - 88.4) ^c^	
**Adacel** ^**®**^^**b**^	66.8 (62.5 - 70.9)	
**Tetavax** ^**®**^ ** or Vacteta** ^**®**^	69.5 (62.6 - 75.8)	
**Tdap (Infanrix** ^**®**^ **, GSK)**		55.7 (46.1 – 64.9) ^d^

^a^ Values are expressed as proportion percentage (95% CI).

^b^ Booster response for Tenivac^®^ and Adacel^®^ defined as a 4-fold rise in antibodies, if the pre-vaccination concentration was equal or below the cut-off value (2.7 IU/mL) and a 2-fold rise in antibody concentration if the pre-vaccination concentration was above the cut-off value (2.7 IU/mL).

^c^ Iranian Td non-inferior to Tenivac^®^ [upper limit of 95% CI for difference (Tenivac^®^ minus Iranian Td) 2.94% < 10%].

^d^ Booster response for Tdap (Infanrix^®^, GSK) is defined as a 4-fold rise in antibody levels if pre-vaccination concentration < 1.0 IU/mL and a 2-fold rise in antibody concentration if the pre-vaccination antibody concentration ≥ 1.0 IU/mL and < 6.0 IU/mL.

Comparing the 4-fold seroconversion rate of the Iranian vaccine (75%) in the current study with the Tenivac^®^ vaccine (84%) showed that the Iranian tetanus vaccine met the non-inferiority criterion and was non-inferior to the Tenivac^® ^vaccine. According to the established non-inferiority criterion, the difference between the upper limit of the 95% confidence interval of the seroconversion rate of Tenivac^®^ minus SCR4 of the Iranian tetanus vaccine was 2.94%, which is less than 10% (Table 4). Additionally, the seroconversion rate, defined as at least a 4-fold increase in antibodies (SCR4), of the Iranian tetanus vaccine (75%) was not inferior to that of the Adacel® vaccine (66.8%) (Table 4). Furthermore, the SCR4 of the Iranian tetanus vaccine (75%) was non-inferior to the SCR4 of the Tetavax^®^ or Vacteta^® ^vaccine (69.5%) (Table 4). 

In the present study, the combined seroconversion (SCR4 and SCR2) rate of the Iranian tetanus vaccine (90%) was compared with the Tdap vaccine (Infanrix^®^, GSK) (55.7%) and showed that the Iranian tetanus vaccine was non-inferior to the Tdap vaccine (Infanrix^®^, GSK).

The multivariate regression method was used to test the correlation between the seroconversion rate in the study population and demographic data. SCR4 was influenced by female gender and age. There was a significant positive correlation between SCR4 and female gender (P < 0.05). In contrast, there was a significant negative effect on SCR4 in people aged 43 years and older. No association was found between other predictors such as concomitant treatment, associated adverse events, BMI, and the period before vaccination with the seroconversion rate.

The correlation between demographic data and the combined seroconversion rates (SCR4 and SCR2) was also tested to determine whether the results of the associations could be modified (Table 5). No association was found between combined seroconversion rates (SCR2 and SCR4) and demographic data (Table 5). 

**Table 5.** Correlation Between Demographic Data and Seroconversion Rates Including the 95% Confidence Interval

**Predictor**s	**Seroconversion, at Least a 4-fold Rise in Antibody Levels**		**A Combined Seroconversion (SCR4 and SCR2)**	
	**Rate (%)**	**P-Value ** ^**a**^	**Rate (%)**	**P-Value ** ^**a**^
**Sex**		0.018		0.284
Male	40 (16.34 - 67.71)		50.0 (26.02 - 73.98)	
Female	60 (32.29 - 83.66)		50.0 (26.02 - 73.98)	
**Medical history**		0.316		0.441
No	80 (51.91 - 95.67)		83.33 (58.58 - 96.42)	
Yes	20 (4.33 - 48.09)		16.67 (3.58 - 41.42)	
**Concomitant medication**		0.071		0.487
No	66.67 (38.38 - 88.18)		72.22 (46.52 - 90.31)	
Yes	33.33 (11.82 - 61.62)		27.78 (9.69-53.48)	
**Related adverse events**		0.065		0.218
No	66.67 (38.38 - 88.18)		61.11 (35.75 - 82.70)	
Yes	33.33 (11.82 - 61.62)		38.89 (17.30 - 64.25)	
**Age (y)**		0.021		0.178
25 - 30	20.0 (4.33 - 48.09)		22.22 (6.41 - 47.64)	
31 - 36	26.67 (7.79 - 55.10)		27.78 (9.69 - 53.48)	
37 - 42	26.67 (7.79 - 55.10)		27.78 (9.69 - 53.48)	
≥ 43	26.67 (7.79 - 55.10)		22.22 (6.41 - 47.64)	
**BMI (kg/m** ^**2**^ **)**		0.258		0.201
18.5 - 24.99	46.67 (21.27 - 73.41)		44.44 (21.53 - 69.24)	
25 - 29.99	26.67 (7.79 - 55.10)		33.33 (13.34 - 59.01)	
≥ 30	26.67 (7.79 - 55.10)		22.22 (6.41 - 47.64)	
**Pre-vaccination period** (y)		0.391		0.863
10 - 14	60.0 (16.34 - 67.71)		38.89 (17.30 - 64.25)	
15 - 29	53.33 (26.59-78.73)		50.0 (26.02 - 73.98)	
≥ 30	6.67 (0.17 - 31.95)		11.11 (1.37 - 34.71)	
**Pre-vaccination antibody level (IU/mL)**		0.137		0.827
≤ 1.7	60.0 (32.29 - 83.66)		50.0 (26.02 - 73.98)	
> 1.7	40.0 (16.34 - 67.71)		50.0 (26.02 - 73.98)	

^a^ Statistical significance determined by multivariate regression.

## 5. Discussion

This study was conducted to evaluate the immune response to a booster dose of the Iranian formulation of the adult Td vaccine in healthy Iranian adults aged 18 - 60 years who had not received the Td vaccine in the past 10 years. The results of this study could be used to generate hyperimmune sera from individuals who received booster doses (10 years later) of the Iranian Td vaccine. The immune response was reflected in post-vaccination seroconversion rates, geometric mean concentrations (GMCs), and increases in antibodies after boosting against tetanus.

The results of this study, in terms of the effectiveness of the Td vaccine available in Iran (manufactured by Razi^®^ Vaccine and Serum Research Institute) as a booster dose, showed that all subjects (100%) were sufficiently immune before booster vaccination, reaching levels of anti-tetanus toxoid antibodies ≥ 0.1 IU/mL. Additionally, 70% of subjects achieved long-term immunity with pre-vaccination levels higher than 1.0 IU/mL, consistent with recently published studies on Tenivac^®^, Adacel^®^, Tetavax^®^, and Vacteta^®^ vaccines (14, 15, 23). All subjects (100%) in the present study achieved post-vaccination levels of anti-tetanus toxoid antibodies ≥ 1.0 IU/mL, which was similar to the results of studies conducted on Tenivac^®^, Adacel^®^, Tetavax^®^, and Vacteta® vaccines (14-17, 23).

A statistically significant increase in anti-tetanus antibody levels from 2.0100 IU/mL (pre-vaccination) to 13.5919 IU/mL (post-vaccination) was observed, similar to the results of Aminzadeh et al.'s study on hemodialysis patients (24). This demonstrated that anti-tetanus antibodies were significantly elevated in subjects following tetanus booster vaccinations.

World Health Organization guidelines define seroconversion as an increase in antibody titer of 2- to 4-fold after vaccination (12). Accordingly, the seroconversion rate in the present study was 75% with a 4-fold increase in antibody titer. The 4-fold seroconversion rate of the Iranian Tetanus vaccine (75%) demonstrated that it was not inferior to the Tenivac^®^ (84%), Adacel^®^ (66.8%), Tetavax^®^, and Vacteta^®^ vaccines (69.5%) (14, 15, 23).

It is noteworthy that the seroconversion result, defined as at least a 4-fold rise in antibody levels (SCR4), of the Iranian tetanus vaccine was compared with the booster response of Tenivac^®^ and Adacel^®.^ For these vaccines, a 4-fold increase in antibody concentration was considered if pre-vaccination concentrations were equal to or below the cut-off value, and a 2-fold increase if pre-vaccination concentrations were above the cut-off value. The cut-off value for tetanus was 2.7 IU/mL (14, 23). The SCR4 result of the Iranian Tetanus vaccine was compared only with the SCR4 result of the Tetavax^®^ or Vacteta^®^ vaccine (15).

The seroconversion rate in this study was calculated as a combination of two- and four-fold increases, similar to the Tenivac^®^ and Adacel^®^ vaccines. Based on WHO guidelines and the Infanrix^®^ vaccine study, 1.0 IU/mL was considered the minimum anti-tetanus antibody concentration before vaccination. This means that for pre-vaccination antibody levels of ≤ 1.0 IU/mL, a 4-fold increase in antibody titer is considered, while for antibody levels of > 1.0 IU/mL, a 2-fold increase in antibody titer is considered (13). This study determined a combined seroconversion rate of 90%. Accordingly, the combined seroconversion rate in the present study (90%) was compared with that of the Infanrix^®^ vaccine (55.7%). The results showed that the Iranian Tetanus vaccine was not inferior to Infanrix^®^. In the Infanrix^®^ vaccine study, the booster dose was administered 10 years after the first booster in adolescence, and the seroconversion rate was calculated as a combination of a 2- or 4-fold increase in tetanus antibody titer based on a minimum concentration of 1.0 IU/mL before vaccination (13).

This study investigated the effect of demographic data, including age, sex, height, weight, BMI, medical history, concomitant medication, vaccination-related adverse effects, pre-vaccination antibody levels, and pre-vaccination period on seroconversion rates, post-vaccination GMCs, and antibody rise after tetanus booster immunization. All subjects had a second blood sample taken 28 days post-vaccination to minimize the possible impact of this interval (post-vaccination period) on the immune response. No demographic data affected the combined seroconversion rate (SCR4 and SCR2), GMC after vaccination, or pre- to post-booster level ratio.

There was no significant relationship found between at least a 4-fold antibody rise and demographic data, except for age and gender. Only two independent variables, age (P-value = 0.021) and female gender (P-value = 0.018), were significant predictors of the seroconversion rate. In terms of age, a significant association was investigated in the age groups of 25 - 30 years, 31 - 36 years, 37 - 42 years, and ≥ 43 years. The 4-fold seroconversion rate was inversely correlated with age, particularly in those 43 years and older (P-value < 0.05). Thus, the 4-fold seroconversion rate declined with increasing age. As age increased, post-vaccination antibody levels decreased precipitously, according to Hainz et al. (25). This steep decline in tetanus vaccination efficacy begins at age 40, with a noted decline in immune responses to tetanus vaccination in adulthood (25). According to Price and Makinodan , antibody responses to most foreign antigens decline with age (26). Older individuals have a lower antibody response to tetanus toxoid (TT) (27, 28). Human serum autologous anti-idiotype antibodies were found to be elevated after TT vaccination by Geha, Saxon, and Barnett (29, 30). According to Arreaza et al., older volunteers showed significantly lower anti-TT antibody responses after a booster dose (31). However, TT booster vaccination resulted in higher levels of auto-anti-Id antibodies in elderly serum (31). Thus, inverse correlations were found between serum anti-TT antibody responses and serum autoantibody levels. This conclusion is consistent with the view that high autoantibody levels in the elderly contribute to impaired antibody responses to TT (31).

Regarding gender, the at least 4-fold increase in anti-tetanus antibody concentrations was 45% in female blood samples and 30% in male blood samples. Therefore, this 4-fold seroconversion rate was more pronounced in women than in men. The study by Fischinger et al. also reported that women tend to have stronger antibody responses and experience more side effects after vaccination compared to men (32). In contrast to this study, Petráš and Oleár reported no significant association between antibody response to booster doses of the tetanus vaccine and volunteer age or gender (15).

As in the Tetavax^®^ and Vacteta^®^ studies, no significant association was found between seroconversion rate and concomitant medications or vaccination-related adverse effects in this study (15). However, unlike the present study, the Tetavax^®^ and Vacteta^®^ study found that the seroconversion rate was dependent on pre-vaccination anti-tetanus antibody concentration, with lower levels predicting a greater immune response (15). Additionally, Petráš and Oleár reported an inverse relationship between the pre-vaccination period and the SCR4 rate, which was not observed in the present study (15). 

The small sample size of this study may have been insufficient to detect any relevant relationships between seroconversion rate, pre-vaccination concentrations of anti-tetanus antibodies, and the pre-vaccination period. Moreover, according to the WHO report, seroconversion rates depend not only on the time between initial and booster doses but also on variables including the number of tetanus toxoid doses received, age at vaccination, and genetics (33).

The present study reported no significant association between the 4-fold seroconversion rate and height, weight, or BMI. According to Petráš and Oleár, obese or overweight subjects had a greater chance than normal-weight subjects of achieving at least a fourfold increase in antibodies (15). However, a 2006 study by Eliakim et al. found a significant decline in tetanus-specific IgG levels in children aged 8 to 17 with BMI above the 85th percentile several years after being vaccinated as infants or children, compared to children with normal body weight (34). Consequently, the lack of a significant association between seroconversion rate and weight or BMI in this study can be explained by the contradictory results of these previous studies.

The present study reported only the common local and systemic side effects of the vaccine. The small sample size (20 volunteers) prevented a reliable estimation of adverse event percentages. Among the side effects reported up to one hour after vaccination, local side effects were more frequent (35%) than systemic (10%). The most frequent local adverse reaction was mild pain at the injection site, with a frequency of 30%.

In the study by Halperin et al., local side effects were most prevalent, with pain at the injection site reported in 84.4 - 87.8% of cases. Similarly, in Halperin et al.'s study, less than 3% of participants reported severe local (grade 3) and systemic complications, which were uncommon (35). The safety results in Halperin et al.'s study were similar to those of the Adacel^®^ vaccine, which were reported in the first 7 days after receiving a booster dose (23, 35). In the Adacel^®^ (Td537) study, pain at the injection site was reported as a frequent adverse event, occurring in 87.1% of cases. Other local adverse reactions, including swelling and redness, were reported at a frequency of 5% each up to one hour after injection in the present study. In contrast, other local reactions in Halperin et al.'s study were reported in 20 – 30% of participants.

The difference between the frequency of injection site pain in this study (30%) and the frequency reported in Halperin et al.'s study (84.4 - 87.8%) and the Adacel^®^ study (Td537) (87.1%) can be attributed to the small sample size in our study, which did not allow for a reliable estimation of the percentage of adverse reactions (23).

Systemic side effects were reported in 10% of volunteers in this study. The most common side effects were lethargy, bruising, hand heaviness, dizziness when changing posture, and sleep disorders. In comparison, at least one systemic side effect was reported by 77% of booster dose recipients in the Adacel^®^ study (Td518). The incidence of other systemic adverse reactions, including headache, muscle weakness, arthralgia, and fever, was zero in the present study. In contrast, other systemic side effects were reported in the study by Halperin et al. between 30 and 40% of participants. The difference in the frequency of side effects reported in this study compared to the Halperin and Adacel^®^ studies may be due to the small sample size in this study, which does not allow for a reliable determination of the percentage of side effects (23).

Adverse events in this study resolved without specific medical intervention within seven days, and no serious adverse events were reported. This is similar to the Adacel^®^ study, in which only 0.8% of serious adverse events were reported (23). No adverse events were reported one week and one month after vaccination. Overall, the studies show that the side effects of tetanus vaccination in healthy adults are mild and tolerable.

In conclusion, the Iranian tetanus vaccine produces both a minimum protective titer and a long-term immune titer in all subjects. The seroconversion rate of the Iranian tetanus vaccine, based on 4-fold and combined criteria (2-fold and 4-fold), was determined to be 75% and 90%, respectively, which is very satisfactory compared to global tetanus vaccines such as Tenivac^®^ and Adacel^®^. Examining the effects of demographic parameters on the seroconversion rate of the Iranian tetanus vaccine showed that only female gender and age 43 and over were significantly associated with the seroconversion rate.

To date, no studies have been conducted on the Iranian tetanus vaccine to assess its suitability for producing concentrations suitable for plasma donation. The purpose of this study was to determine the proportion of people eligible to donate plasma for hyperimmune plasma manufacturing. Based on this and previous studies, 100% and 45% of individuals are eligible for plasma donation based on minimum (2 IU/mL) and maximum (10 IU/mL) anti-tetanus antibody titers, respectively. Overall, given the mild and well-tolerated adverse events and appropriate immunological response, a single dose of the Iranian tetanus vaccine as a booster in healthy adults aged 18 - 60 years who had not received any vaccine against tetanus and diphtheria in the last 10 years appears to be sufficient and effective for producing concentrations suitable for plasma donation. 

As there are no manufacturers of hyperimmune anti-tetanus plasma in Iran, the results and data from this study may be valuable to those considering such production. However, given the limited number of subjects in our study, further studies on the seroconversion rate of the Iranian tetanus vaccine on a larger scale and with more samples should be conducted in healthy adults. This would ensure that the data analysis is both statistically more valuable and reliable for use in studies related to the production of hyperimmune tetanus serum in Iran.

Additionally, no studies have been conducted on tetanus antibody levels even 10 years after the initial vaccination with the Iranian tetanus vaccine. Therefore, one limitation in the implementation of the above plan was recruiting volunteers to participate. Another important limitation was the need for a leadership platform and facilities such as the presence of a doctor, a suitable place for sampling, a study management team, and the implementation of the anti-tetanus antibody ELISA test. The high cost of the Abnova^®^ KA5796 kit and the cessation of its production by the manufacturer were also significant limitations. Due to these constraints, the current research was conducted with a small number of samples and in a preliminary manner.

A company that produces hyperimmune serum can easily use this information to produce hyperimmune sera for tetanus. Ten years after the first vaccination with other vaccines widely used around the world, several studies on the level of tetanus antibodies have been carried out. However, due to the biological nature of tetanus vaccines and their inherent heterogeneity, the results of the Iranian vaccine cannot be accurately generalized. The level of tetanus antibodies 10 years after the Iranian tetanus vaccination can also be used for policymaking. According to some studies, tetanus vaccines can provide protection for up to 30 years. Therefore, conducting the mentioned study may influence Iran's tetanus vaccination policy.

Future studies should consider the complete history of previous tetanus vaccinations, including the age at which the vaccine was administered and the number of doses received, as possible factors affecting seroconversion rates and how the antibody response is related to these factors. Unfortunately, these two factors were not considered in this study based on previous similar studies. Additionally, a study with a larger sample size is recommended to evaluate the hypothesis that there is an inverse relationship between seroconversion rate and age over 43 years, which has not been reported in any other study.

Since the present study was a preliminary investigation of healthy volunteers, many possible influencing factors were not examined. For example, smoking history, as well as host factors such as genetics and immune system defects, may play a crucial role in the seroconversion rate and antibody responses. It is also recommended to conduct a study with a larger number of volunteers to determine if there is a relationship between the seroconversion rate, pre-vaccination concentration, and the time interval since the last vaccination.

## Data Availability

The dataset presented in the study is available on request from the corresponding author during submission or after publication.
